# Supplementary material for: Putting Policy into Practice: How Three Cancer Services Perform against Indigenous Health and Cancer Frameworks
Source: Int J Environ Res Public Health. 2022 Jan 6;19(2):633. doi: 10.3390/ijerph19020633 (PMC8775789; doi:10.3390/ijerph19020633)
Supplement: Supplementary file 1 [file ijerph-19-00633-s001.zip › ijerph-1502550-supplementary.pdf]

Supplement S1: Summary of progress in Service B since interviews were conducted

After semi-structured interviews were conducted at Service B, the service developed and endorsed an Aboriginal Cultural Safety Action Plan (2019-2022) (the Plan) outlining action items for review and improvement. The Plan covers the Aboriginal National Standards and eight key action areas, as determined by the Department of Health and Human Services, aligning the standards to the new fixed grant funding model, which superseded the Aboriginal WEIS funding model. The new model determines funding based on an annual program of Aboriginal cultural safety improvement works assessed via an annual reporting model. The grant supports the service's Aboriginal Hospital Liaison Officer (AHLO, 1.0FTE) and Aboriginal Support Project Officer (ASPO, 1.0FTE).

Since its launch, staff have steadily progressed through the Plan. Plan-related activity ensured the service met the criteria of the National Safety & Quality Health Service Standards (NSQHS) accreditation cycle held in June 2021. The remainder of this supplemental appendix provides an overview of progress as of October 2021.

Aboriginal and/or Torres Strait Islander Advisory Committee

- Reviewed and refreshed the Aboriginal and/or Torres Strait Islander Advisory Committee (AAC) membership and respective Terms of Reference.
- The AAC prioritised the following three projects for implementation in 2021: 1) Patient Identification, 2) Failed to Attend, and 3) Discharge Planning. All projects are underway

Reconciliation Action Plan 2021-2023 (RAP)

- Established and implemented the Reconciliation Action Plan (RAP) project. This included the establishment of a RAP Working Group, RAP Champions Network of over 50 people and derived from this, the four RAP key theme sub-working groups.
- Reviewed and updated the service's Reconciliation Action Plan (RAP). The drafting process was facilitated by Yorta Yorta woman, Karen Milward from Karen Milward Consultancy Services. Karen convened and facilitated a RAP development workshop of both First Nations Peoples and non-Indigenous people. The draft RAP was reviewed extensively and finer detail was applied and updated to deliverables to ensure criteria as set by Reconciliation Australia (RA) were met. The draft was registered with RA in March 2021 and, after several rounds of feedback, conditional endorsement was provided in October 2021. The conditionally endorsed RAP, with incorporated visual design (described next), will be launched at the service's AGM in December 2021.
- An Aboriginal visual identity project was developed in partnership with Marcus Lee Designs, a local First Nations designer. Marcus was engaged to explore and design the visual identity that would reflect the service's commitment to Reconciliation. The concept design process included participation from the AAC, RAPWG, and focus group sessions with First Nations Peoples, staff and external agencies/stakeholders to explore and determine the visual identity

Staffing

- Recruited ASPO to support the roll-out of the RAP and other projects as determined by the AAC.
- Introduced the 6-month Aboriginal Summer Internship program. The inaugural incumbent undertook a patient experience literature review supported by relevant departments, which will guide and inform the service's ongoing approach to improving patient experiences.

Partnerships

- Initiated a conversation with Victorian Aboriginal Community Controlled Health Organisation (VACCHO) to re-sign the Memorandum of Understanding which expired in Aug 2020.
- Commenced a discussion with the Victorian Aboriginal Health Service (VAHS) to strengthen referral pathways for First Nations people.

Cultural Awareness, Safety and Related Activities

- Launched the Aboriginal and Torres Strait Islander Cultural Awareness Training package. The package comprises three modules for all staff plus one extra module for managers. Completion rates for 'All Staff Modules' between February and October 2021 were 65%. Completion rates for the 'Manager Module' between February and October 2021 were 47%.
- Contracted VACCHO to deliver Aboriginal Cultural Safety training sessions. The sessions ran from 9am to 2pm and due to COVID restrictions, were offered via Zoom. Between April and June 2021, 46 staff attended five scheduled sessions. Feedback from staff suggested the timing of sessions were limiting and unfeasible due to their roles in heavy patient facing environments.
- Explored other options for flexible and targeted training based on preliminary findings from the AAC directed projects and staff feedback on the VACCHO training. As a result, the service offered a 90-minute "*How to ask the question training*", which incorporated important considerations for cultural safety. This training was delivered by Girraway Ganyi Consultancy via Zoom. In October 2021, 42 staff attended two scheduled sessions.
- Redeveloped collateral to promote information to patients in regards to "Why we ask, Are you of Aboriginal and/or Torres Strait Islander origin?'.
- Spotlighted the new mandatory Aboriginal and/or Torres Strait Islander Cultural Awareness Training during NAIDOC 2020 Week. The virtual event included a Welcome to Country, a focused presentation on the Cultural Safety e-Learning program and a dance performance by the Djirri Djirri dance company. Plans for NAIDOC Week 2021 were curtailed due to COVID-19 restrictions. To highlight progress on the service's RAP, three of the service's RAP Champions and related materials were promoted via the intranet in place of face-to-face events.
- Established a Yarning Circle program in response to requests from First Nations patients to the AHLO. The first Yarning Circle session was launched in July 2021 to coincide with NAIDOC Week 2021 celebrations. Further sessions took place in August and September 2021.
- Procured culturally accepted furnishings and musical instruments, as a sense of visual and welcoming environment is paramount to cultural safety for First Nations Peoples. These affects are used in spaces where First Nations Peoples do their 'business', such as the Yarning Circle.

Information Resources

- Development of two resource booklets: 1) *Wominjeka*: a resource to support and provide information to First Nations Peoples that includes available services, and information about the importance of discharge planning, keeping appointments and how to provide feedback to the service, and 2) *Staff Guide*: how to engage with Aboriginal and/or Torres Strait Islander Peoples. This guide provides staff with some key information to assist their understanding of cultural safety, how to ask the identification question, who to contact for assistance in supporting Aboriginal patients/families, dedicated First Nations services, assumptions that should never be made, Aboriginal language and Acknowledgement to Country. Both booklets will be launched at the service's Annual General Meeting in December 2021
